# White matter tract-specific quantitative analysis in multiple sclerosis: Comparison of optic radiation reconstruction techniques

**DOI:** 10.1371/journal.pone.0191131

**Published:** 2018-01-17

**Authors:** Chenyu Wang, Alexander Klistorner, Linda Ly, Michael H. Barnett

**Affiliations:** 1 Sydney Neuroimaging Analysis Centre, Sydney, New South Wales, Australia; 2 Brain and Mind Centre, University of Sydney, Sydney, New South Wales, Australia; 3 Department of Ophthalmology, Save Sight Institute, University of Sydney, Sydney, New South Wales, Australia; 4 Australian School of Advanced Medicine, Macquarie University, Sydney, New South Wales, Australia; Texas A&M University, UNITED STATES

## Abstract

The posterior visual pathway is commonly affected by multiple sclerosis (MS) pathology that results in measurable clinical and electrophysiological impairment. Due to its highly structured retinotopic mapping, the visual pathway represents an ideal substrate for investigating patho-mechanisms in MS. Therefore, a reliable and robust imaging segmentation method for in-vivo delineation of the optic radiations (OR) is needed. However, diffusion-based tractography approaches, which are typically used for OR segmentation are confounded by the presence of focal white matter lesions. Current solutions require complex acquisition paradigms and demand expert image analysis, limiting application in both clinical trials and clinical practice. In the current study, using data acquired in a clinical setting on a 3T scanner, we optimised and compared two approaches for optic radiation (OR) reconstruction: individual probabilistic tractography-based and template-based methods. OR segmentation results were applied to subjects with MS and volumetric and diffusivity parameters were compared between OR segmentation techniques. Despite differences in reconstructed OR volumes, both OR lesion volume and OR diffusivity measurements in MS subjects were highly comparable using optimised probabilistic tractography-based, and template-based, methods. The choice of OR reconstruction technique should be determined primarily by the research question and the nature of the available dataset. Template-based approaches are particularly suited to the semi-automated analysis of large image datasets and have utility even in the absence of dMRI acquisitions. Individual tractography methods, while more complex than template based OR reconstruction, permit measurement of diffusivity changes along fibre bundles that are affected by specific MS lesions or other focal pathologies.

## Introduction

Precise localisation of focal pathology to function-specific regions within the central nervous system (CNS) is a critical step toward defining biomarkers of MS disease activity and progression [1,2].

The visual pathways constitute an ideal model for probing patho-mechanisms of multiple sclerosis (MS) in vivo since structural and functional properties of the visual system, which is frequently affected by MS pathology, can be assessed by a number of objective, quantitative techniques [3,4]. In the visual system, the retina, optic nerves, optic chiasm, optic tract, and visual cortex can be relatively easily differentiated from surrounding structures, however segmentation of the lateral geniculate nuclei (LGN) and optic radiation (OR) is challenging.

The ORs are a pair of dense white matter (WM) fibre bundles comprising axons that originate from neurons located in lateral geniculate nucleus (LGN) and extend to the calcarine cortex [5–8]. Axons within the OR are situated adjacent to the lateral ventricle in both hemispheres and project in a primarily anterior-posterior orientation from the LGN to the visual cortex. We have previously shown that the majority of subjects with MS have lesions within the OR[9]. However, the OR is poorly identified on conventional T1/T2-weighted images. While susceptibility-weighted imaging (SWI) [10] and phase difference enhanced imaging (PADRE) [11] techniques, which exploit subtle magnetic heterogeneity between the OR and its surrounding white matter fibre structures, more reliably delineate the tract, quantitative techniques based on visualisation of tissue contrast using these sequences have not been validated in MS, where both focal (lesional) pathology and reduced fibre density (particularly in Meyer’s loop and subcortical regions) may directly impact segmentation quality.

Currently, diffusion MRI (dMRI)-based tractography represents the principal technique for mapping human WM fascicles. Application of dMRI to the visual pathways has yielded successful OR segmentation using both deterministic tractography (DT) [12–16] and, more recently, probabilistic tractography (PT) [17–28]. However, there has been also limited application of tractography algorithms to MS cohorts. DT and PT algorithms were developed and validated primarily in healthy controls and their performance in the presence of focal WM pathology is suboptimal [29–31]. Unlike space occupying lesions (i.e. tumour or cyst), in which reconstructed fibre paths are expected to travel around the relevant pathology, inflammatory demyelination within tracts does not likely alter expected location of surviving axons, despite considerable alteration of the local structural environment. As measured by dMRI, inflammatory demyelination commonly reduces fractional anisotropy, which, in turn, hinders conventional tractography results.

Therefore, template-based tract reconstruction may be an alternative approach to delineate white matter fiber tracts in patients with MS [30–32]. Reference templates can be derived either from post-mortem dissections [8] or dMRI-based tractography in healthy controls [30,32]. Template-based approaches also permit tract-specific analysis in the absence of subject-specific diffusion data, simplifying post-processing pipelines and eliminating the need for advanced image acquisitions. However, brain volume loss, dynamic ventricular enlargement, and lesion activity (newly appearing/disappearing, enlarging/shriking) in MS critically impacts the mapping of template-subject ROIs based on transformation matrices estimated by linear/non-linear co-registration[30,31]. Additionally, comparability between tractography-based and template-based tract specific analysis in MS has not been also been thoroughly investigated, which is an important information for choosing the proper approach to investigate microstructure changes along white matter fibres.

Therefore, in this study we quantitatively compared the effect of optimised OR reconstruction techniques, namely individual probabilistic tractography (PT-OR), normal control-based template reconstructed OR (Template-OR), and Jülich histological atlas-based template reconstructed OR (Histology-OR) [8] on MS-associated measures of OR pathology.

## Materials and methods

Thirty-five healthy participants (25 female, 10 male, mean age 36.8 years, SD = 14.62) and seventy MS subjects with relapsing-remitting MS (45 female, 25 male, mean age 41.07 years, SD = 10.44) were included in the study. The study was approved by the human research and ethics committee at the University of Sydney, Sydney, Australia. All participants gave written informed consent.

MRI data was acquired from all subjects with a 3.0 Tesla GE MR750 scanner (General Electric, Milwaukee, WI, USA), using an 8-channel head coil. For each exam, whole brain 64-directions diffusion-weighted imaging was acquired with 2 mm isotropic resolution (TR/TE = 8325/86 ms, b = 1000 s/mm^2^, number of b0s = 2). Additionally, whole brain sagittal IR-FSPGR (TR/TE/TI = 7.2/2.7/450 ms, 1 mm isotropic acquisition matrix, FOV = 256 mm) and sagittal FLAIR CUBE (TR/TE/TI = 8000/163/2180 ms, slice thickness 1.2 mm, acquisition matrix: Freq. × Phase = 256 × 244) were collected.

### Reconstruction of optic radiation using probabilistic tractography

All diffusion weighted imaging (DWI) was converted from DICOM to NIFTI format using the dcm2nii tool from MRIcron Toolbox (v.06/06/2013, http://people.cas.sc.edu/rorden/mricron/dcm2nii.html). DWI was corrected for motion and eddy-current distortion in FSL (FMRIB Software Library; www.fmrib.ox.ac.uk/fsl). EPI susceptibility distortion was then corrected by nonlinear co-registration to T1-weighted structural imaging using ANTS (Advanced Normalisation Tools, http://picsl.upenn.edu/software/ants) after non-brain tissue was removed using BET (Brain Extraction Tool, FSL). Tensor reconstruction and co-registration with T1-weighted imaging were performed in MrDiffusion (MrVista, Stanford University, http://web.stanford.edu/group/vista/cgi-bin/wiki/index.php/MrVista).

**Fig 1:**
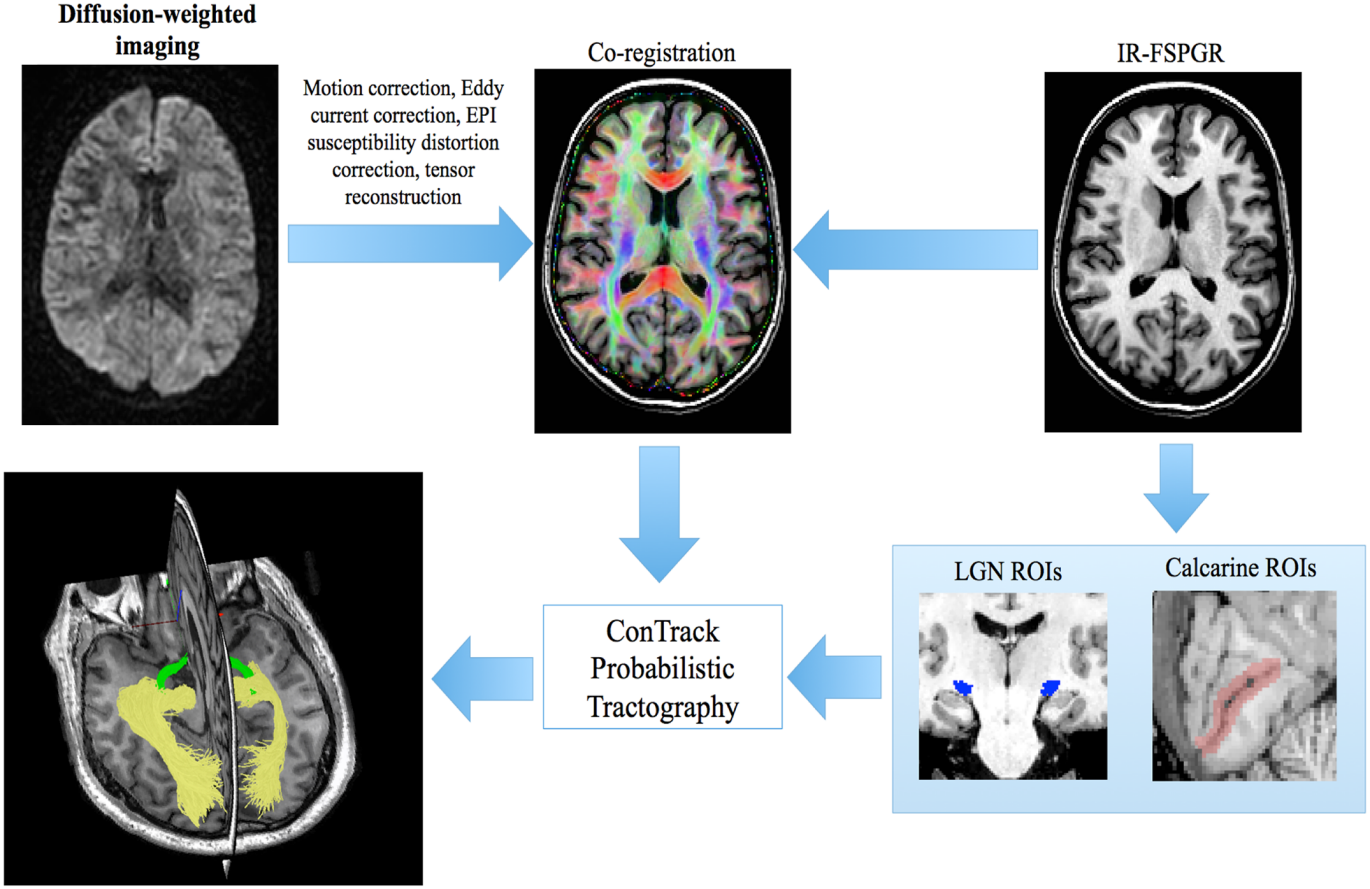
Optic radiation reconstruction pipeline using probabilistic tractography with ConTrack [33].

Seeding points at the LGN and calcarine sulcus were created manually for the reconstruction of OR using ConTrack PT [33] (Fig 1). To identify the LGN, which is nearly invisible on structural T1-weighted images, optic tract fibres were followed from the optic chiasm using DT (a 10 mm ROI placed on the optic chiasm was used to seed the deterministic algorithm). The position of the LGN was inferred by the termination of optic tract fibres, at which point a circular ROI (diameter 7 mm) was placed. An occipital cortex ROI covering the calcarine sulcus was manually drawn on the high resolution T1 structural image in each hemisphere using the editable ROI function of FSLVIEW software. All manually placed ROIs were reviewed by two experts (SK, CW).

ConTrack [33] PT was used to reconstruct bilateral OR for each participant. Cerebellum was removed from tractography prior masks automatically to ensure OR fibre tracking was confined to the cerebrum; and a segment of corpus callosum (CC) 3 mm in width, centred on the mid-sagittal plane, was removed to avoid propagation of tracts into the contralateral hemisphere. This pipeline was implemented by applying a constraining mask that was warped nonlinearly from ICBM 2009a standard brain with the cerebellum and CC removed. Then, an initial 75000 fibres were generated between ipsilateral seeding points. Of these, 30000 fibres were selected by thresholding to those with the highest tracking scores [33]. Finally, all reconstructed ORs were reviewed by two experts (SK and CW) to ensure anatomic plausibility and refined where necessary (Fig 1).

### Construction of optic radiation template from healthy controls

Following removal of non-brain tissue (Brain Extraction Tool, FMRIB software Library), T1-weighted images for each of the 35 HCs in the study were non-linearly co-registered to ICBM 2009a standard brain using ANTS to obtain the deformation maps. Individual PT-OR in HCs were binarised, projected into ICBM standard space and then averaged voxel-wise to construct the OR probability template, which was normalised according to the likelihood of a voxel being present in the HC OR (0 = voxel present in 0/35 HC ORs; 1 = voxel present in 35/35 HC ORs).

To facilitate visual comparison of individual binarised PT-ORs with the constructed OR template, a binarised OR template was produced from the probability OR template. The OR probability template was thresholded at different levels (assigning a value of 0 to voxels below the probability thresholding value) and compared with PT-OR reconstructions using a Dice Similarity Coefficient (DSC) to generate the optimal probability threshold prior to binarisation. Briefly, the OR probability map in ICBM standard space was thresholded from 1% to 99% with 1% increments; all thresholded OR templates were then individually mapped onto HC brains in subjects’ native space using inversed deformation maps, and compared with individual PT-OR using the DSC. The threshold that produced the largest mean DSC between Template-OR and PT-OR among HC subjects was used to binarise the OR probability map and generate the final OR binary template in ICBM standard space.

### Whole brain volume assessment, thalamus masks and optic radiation lesion analysis in subjects with multiple sclerosis

For all subjects with MS, FLAIR images were co-registered with T1-weighted structural images using FLIRT from FSL Toolbox with 6-parameters transformation. Whole brain T2 lesion masks were semiautomatically segmented from co-registered FLAIR scans by a trained analyst using ITK-SNAP (http://www.itksnap.org/pmwiki/pmwiki.php). Prior to the calculation of whole brain volume and the extraction of WM partial volume maps by using SIENAX (FSL) [34], the “lesion-filling” tool (FSL) was applied to the T1-weighted images using the T2 lesion masks to avoid tissue misclassification due to WM pathology [35]. “Lesion-filled” and skull-stripped T1-weighted brain images were then non-linearly co-registered with ICBM 2009a standard brain using ANTS to derive reference-subject space deformation matrices. The OR template constructed from HCs (Template-OR) and from the Jülich histological atlas (Histology-OR) were then non-linearly mapped to individual subjects’ space.

Both the Template-OR and Histology-OR masks are probability weighted, while the PT-OR is derived from individual tractography and presented as a binarised mask. Prior to OR related calculations, the thalamus was excluded by applying masks derived from FSL/FIRST analysis. The calculation of all OR related measurements were performed in the subjects’ native space, unless otherwise specified.

T2 lesion masks were overlaid with the three OR masks (PT-OR, Template-OR and Histology-OR) to calculate OR lesion volumes and other OR lesion related metrics. For probability-weighted OR masks (constructed from either the HC dataset as described, or from the Jülich histological atlas), the OR T2 lesion volume was calculated with weighted probability as follows:

$$V=r\sum_{i=1}^{n}v_{i}\omega_{i}$$

Where *ν*_*i*_ is the value of the voxels in the whole brain binarised T2 lesion masks (which is 1 in this case); *ω*_*i*_ is the value of the corresponding voxel in the probability-weighted OR masks; and r is the resolution of the voxel.

OR voxels that overlapped T2 lesions were classified as lesional OR, and OR voxels free of visible T2 pathology as non-lesional OR. We propose lesional OR as a window for monitoring de- and re-myelination and axonal loss in focal acute/chronic lesions; and non-lesional OR as a platform to investigate microstructural changes in normal appearing white matter in MS.

### Optic radiation diffusivity analysis

Co-registered axial diffusivity (AD), radial diffusivity (RD), mean diffusivity (MD) and fractional anisotropy (FA) maps were generated for each subject. OR masks, including entire OR, lesional and non-lesional OR were co-registered and overlayed with diffusivity maps to calculate the probability weighted mean as follows:

$$\overset{-}{D}=\frac{\sum_{i=1}^{n}D_{i}\omega_{i}}{\sum_{i=1}^{n}\omega_{i}}$$

Where *D*_*i*_ is the value of the voxel in the diffusivity map and *ω*_*i*_ is the value of the corresponding voxel in the probability-weighted OR masks

### Statistics

Statistical analysis was performed using SPSS 22.0 (SPSS, Chicago, IL, USA). One-way ANOVA was used to assess differences between OR metrics estimated by different approaches, while Wilcoxon Signed Rank test was used for pairwise comparison of data that was not normally distributed. Statistical agreement between methods was assessed with Pearson’s correlation coefficient (r) and Bland-Altman plots. P values less than 0.05 were considered statistically significant.

## Results

### Construction of the optic radiation template

**Fig 2:**
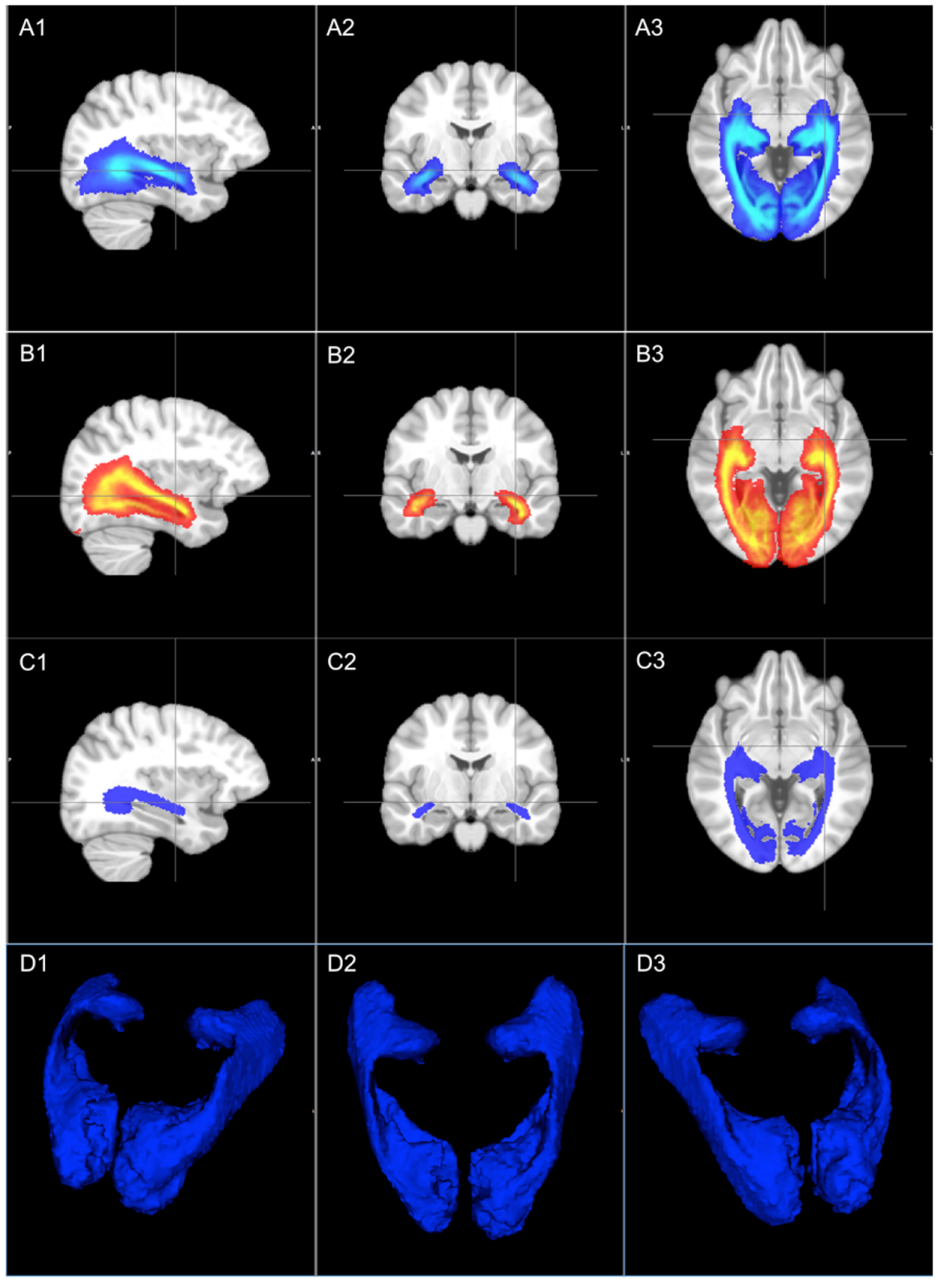
(A1-A3) Template-OR constructed from 35 healthy controls using ConTrack probabilistic tractography [33], represented as a probability map; (B1-B3) OR probability map derived from the Jülich histological atlas [8] (Histology-OR); (C1-C3) OR binary template thresholded at 0.32 from probability map shown in A; (D1-D3) 3D view of OR binary template.

Reconstruction of the OR using PT was performed in all HC and MS subjects. Table 1 shows demographics and baseline measurements for MS patients included in the study. The template-OR constructed from 35 HCs is represented as a probability map in (Fig 2A1–2A3). Topographic conformity with the Jülich histological atlas [8] was visually confirmed (Fig 2B1–2B3); in particular, Meyer’s loop was clearly defined in the Template-OR (Fig 2D1–2D3). The estimated volume of Template-OR and Histology-OR was 31.78 mL and 27.35 mL respectively, relative to a whole standard brain volume of approximate 1511 mL in ICBM reference space.

**Table 1:** MS subject characteristics.

Variable	All MS subjects (N = 70)
Sex, n (%)	
Female	45 (64.3)
Male	25 (35.7)
Age, mean ± SD, years	41 ± 10.44
Disease duration, mean ± SD, years	5 ± 4.78
SIENAX Normalised BV, mean ± SD (min–max), mL	1510 ± 89.0 (1260–1698)
Normalised PT-ORv, mean ± SD (min–max), mL	28 ± 7.3 (12–41)
Normalised Template-ORv, mean ± SD (min–max), mL	32 ± 3.4 (22–40)
Normalised Histology-ORv, mean ± SD (min–max), mL	27 ± 2.8 (19–34)
PT-ORv/BV, mean ± SD (min–max), %	1.9 ± 0.46 (0.9–3.0)
Template-ORv/BV, mean ± SD (min–max), %	2.1 ± 0.17 (1.6–2.5)
Histology-ORv/BV, mean ± SD (min–max), %	1.8 ± 0.14 (1.3–2.1)
T2 lesion volume, mean ± SD (min–max), mL	7 ± 9.1 (0.1–60)

SD, standard deviation; BV, brain volume; ORv, optic radiation volume; Normalised OR volumes were calculated by multiplying the absolute OR volume with the scaling factor derived from SIENAX, which was estimated though affine-registration with MNI152 spacing using the skull image.

The binarised template-OR was derived from the probability weighted OR template, optimised using a threshold of 0.32, which produced a maximum average DSC (0.72) with individual PT-OR in HCs (S1 File). Fig 2C1–2C3 shows the binarised OR template, and Fig 2D1–2D3 shows the 3D reconstruction in ICBM space.

**Fig 3:**
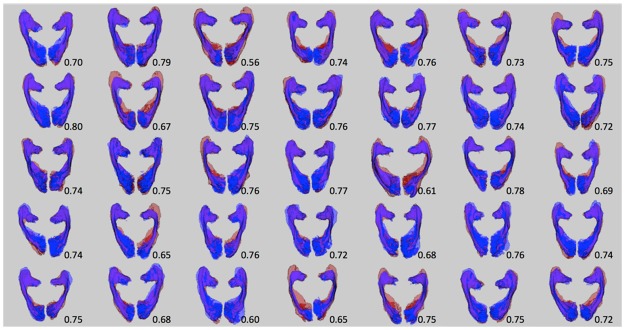
Comparison of Template-OR (Red) and PT-OR (Blue) for all 35 healthy controls in subjects’ native space. Prior to non-linearly mapping the Template-OR from ICBM space to the subjects’ native space, the probability weighted template-OR (in ICBM space) was thresholded at 32% and binarised. DSC are labelled at the bottom right corner of each pair of ORs.

Fig 3 shows the individual PT-OR (Blue) and template-OR (Red) in HC subjects’ space and the individual DSCs between the two OR methods.

### Optic radiation reconstruction in subjects with MS

**Fig 4:**
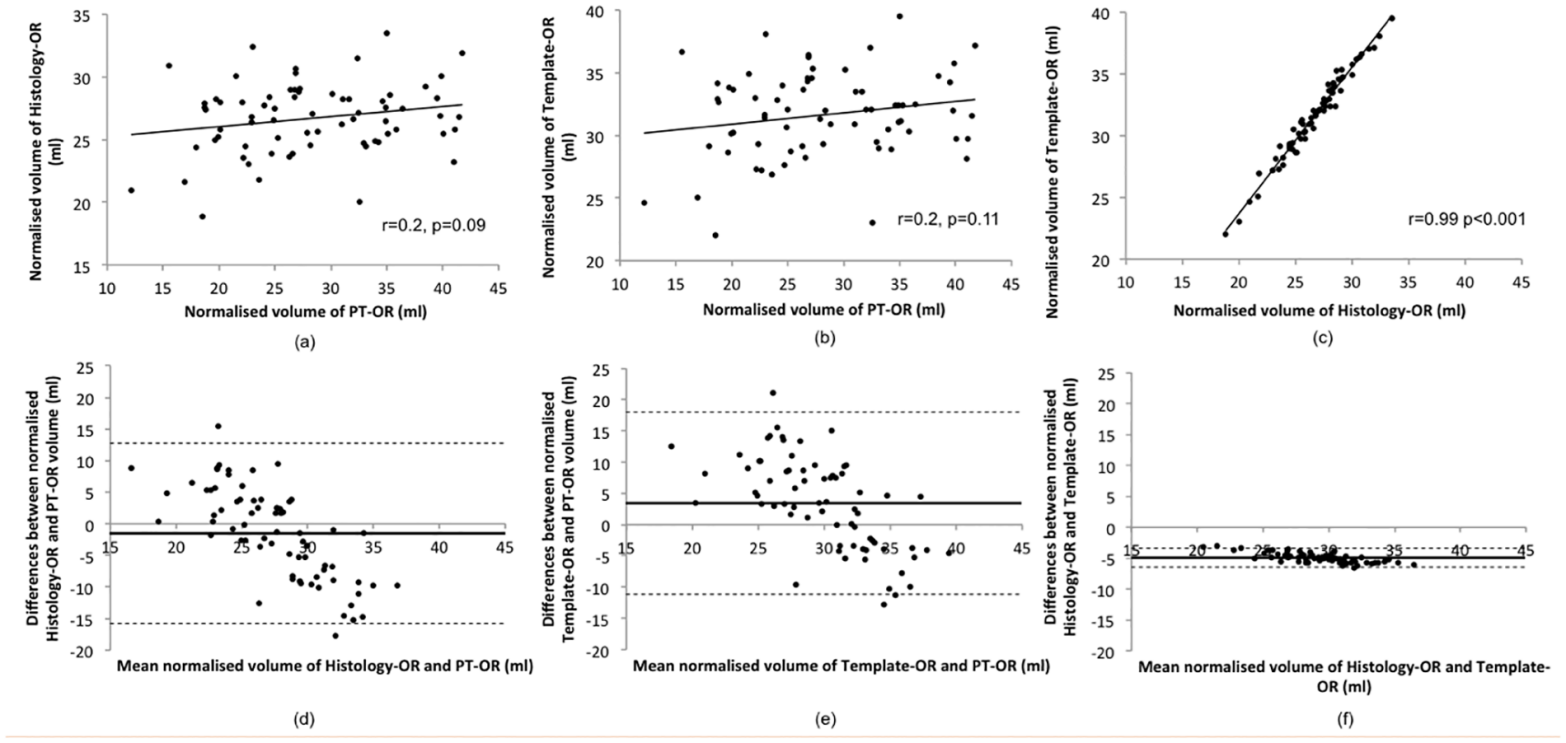
Comparison of OR volume estimation by PT-OR, Template-OR and Histology-OR.

In subjects with MS, the mean volume of Template-OR was significantly larger than PT-OR (31.64 vs 28.22 mL, oneway ANOVA, post-hoc Tukey HSD test, p<0.001) and Histology-OR (31.64 vs 26.70 mL, oneway ANOVA, post-hoc Tukey HSD test, p<0.001), after normalisation by skull size. There was no significant difference between Histology-OR and PT-OR volume (26.70 vs 28.22 mL, oneway ANOVA, post-hoc Tukey HSD test, p = 0.17). Pearson correlations showed a high agreement between Template-OR derived and Histology-OR derived volume (r = 0.99, p<0.001), while neither correlated with tractography-based PT-OR volume (Fig 4). Bland-Altman plots also demonstrated consistency between Template-OR and Histology-OR at both large and small OR volumes, but neither with PT-OR (Fig 4).

**Fig 5:**
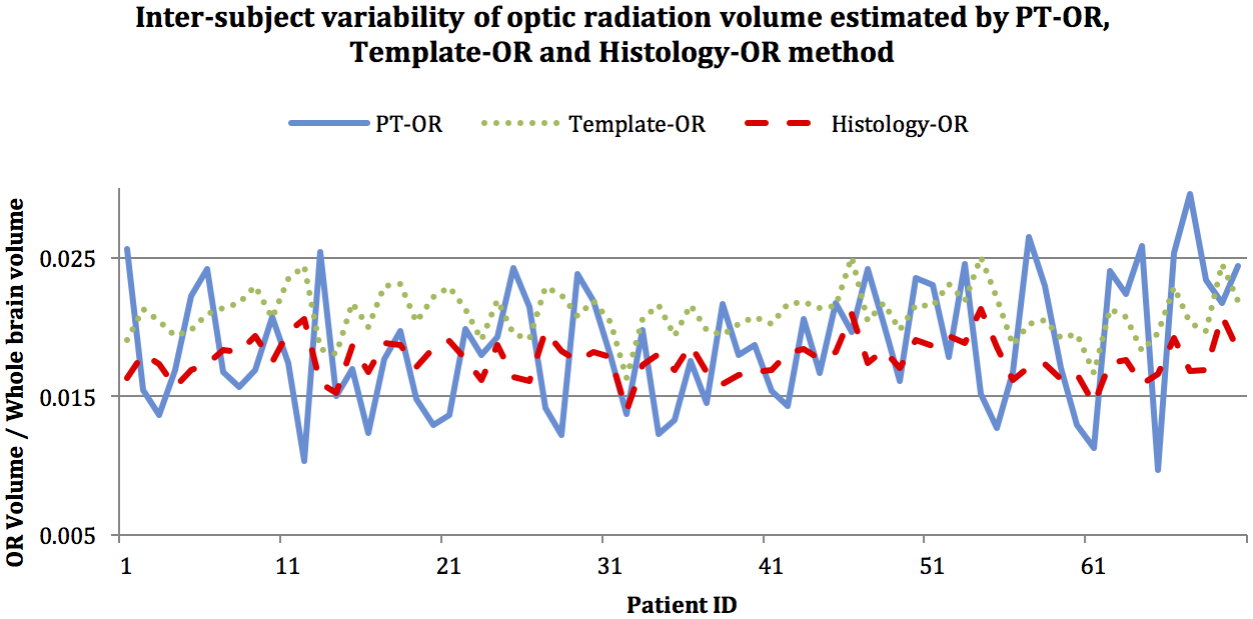
Inter-subject variability of OR volume estimated by PT-OR (blue), template-OR (Green) and Histology-OR.

The standard deviation for Template-OR and Histology-OR was considerably lower compared with PT-OR, indicating considerably lower inter-subject variability (from 26% in PT-OR to 10.79% in Template-OR and 10.61% in Histology-OR). To minimise the effect of methodology-related variability, we normalised OR volume by subjects’ brain volume rather than skull size (to account for MS-related brain atrophy), which further reduced the inter-subject variability coefficient for template-based techniques to 8.3% in Template-OR and 8% in Histology-OR, while PT-OR variability remained as high (24.6%) (Fig 5).

The mean ± SD (min–max) dice similarity coefficient between methods for OR delineation were 0.58 ± 0.053 (0.42–0.67), 0.36 ± 0.044 (0.25–0.44) and 0.41 ± 0.007(0.39–0.43) for PT-OR vs. Template-OR, PT-OR vs. Histology-OR and Histology-OR vs. Template-OR, respectively.

We next investigated the association between OR volume and conventional measures of disease burden in MS, including normalised brain volume (NBV) and total brain lesion volume. As expected, template- and histology-based OR volumes correlated well with NBV (r = 0.655, p<0.001 and r = 0.665, p<0.001, respectively), while the association with PT-OR volume was less robust (r = 0.372, p = 0.002). In addition, total brain lesion volume significantly correlated with Template-OR and His-OR volume (r = -0.612, p<0.001) (r = -0.611, p<0.001), while no association was found with PT-OR volume (r = -0.155, p = 0.201).

### Optic radiation lesion volume estimation in subjects with MS

**Fig 6:**
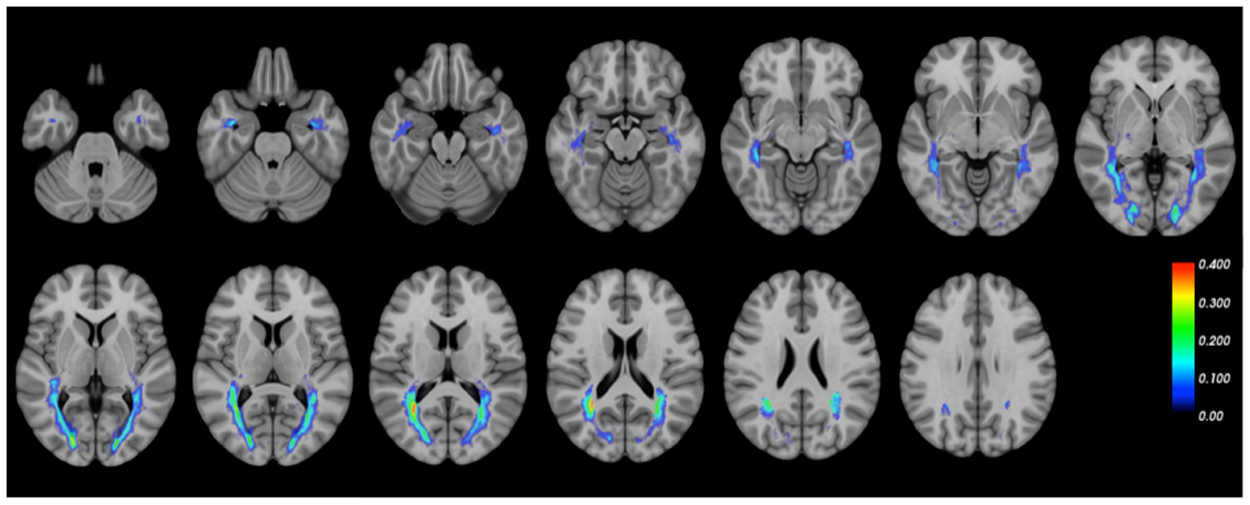
Lesion distribution along the optic radiation in subjects with multiple sclerosis.

**Fig 7:**
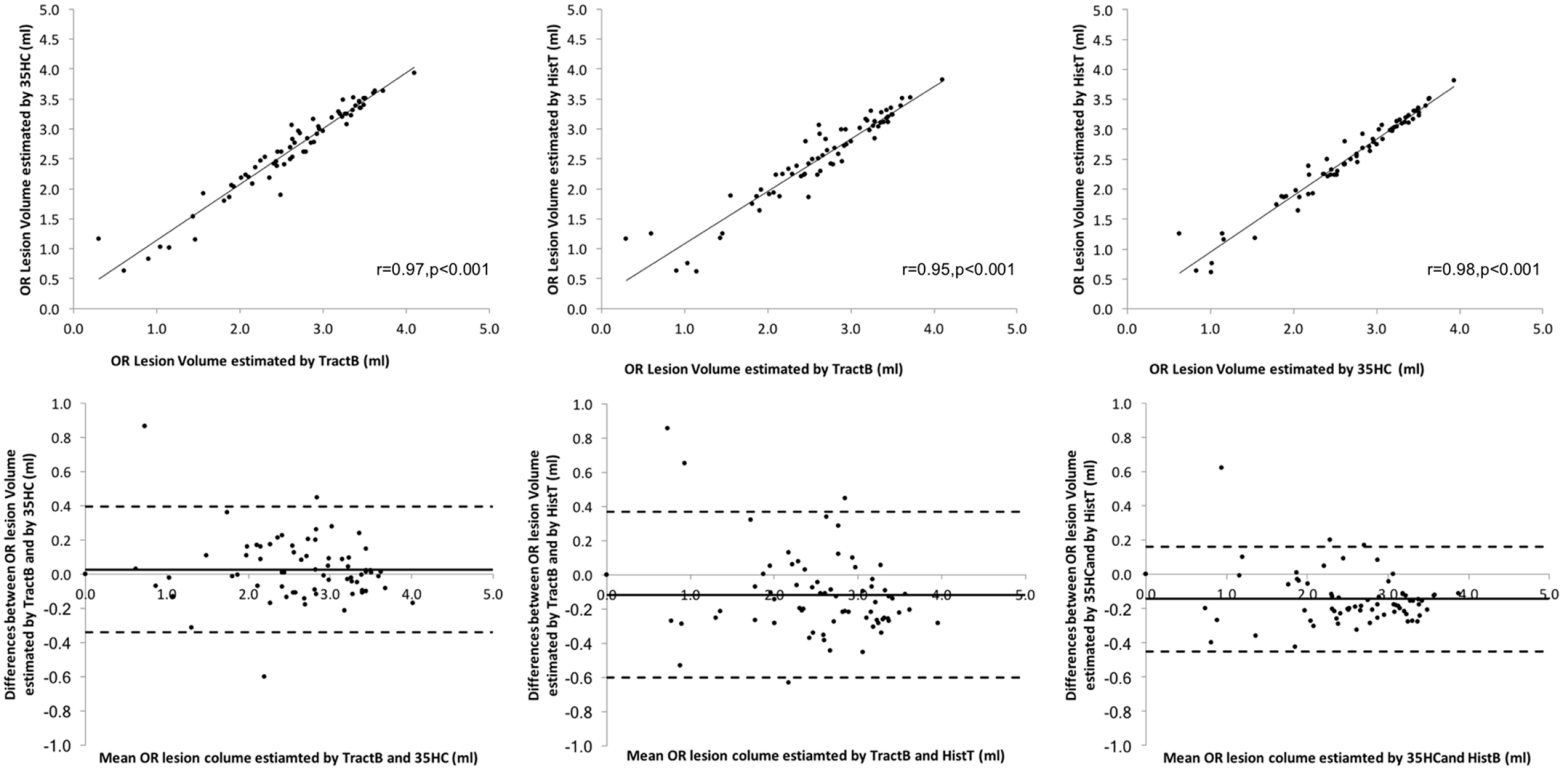
Comparison of OR lesion volume estimated by PT-OR, Template-OR and Histology-OR. The total lesion volume is shown as logarithmic values.

Fig 6 shows the lesion distribution along the optic radiation of 70 MS subjects. Lesions were more frequent in the middle part of the OR adjacent to the lateral ventricle, compared with the anterior and posterior parts of the OR. Wilcoxon Signed Rank test was conducted to compare the OR lesion differences between three methods. There was no statistically significant difference found (p = 0.723) between lesion volume estimated by PT-OR (median 0.482ml) and Template-OR (median 0.581ml), but both methods produced larger lesion volumes than those derived from Histology-OR approaches (median 0.368, p<0.001). There was a high correlation between the volume of OR lesions estimated by the three methods (Fig 7).

The differences between PT-OR and Template-OR were consistent at all lesion volume levels, while Histology-OR tended to underestimate OR lesion volume in MS subjects comparing to PT-OR and Template-OR.

**Fig 8:**
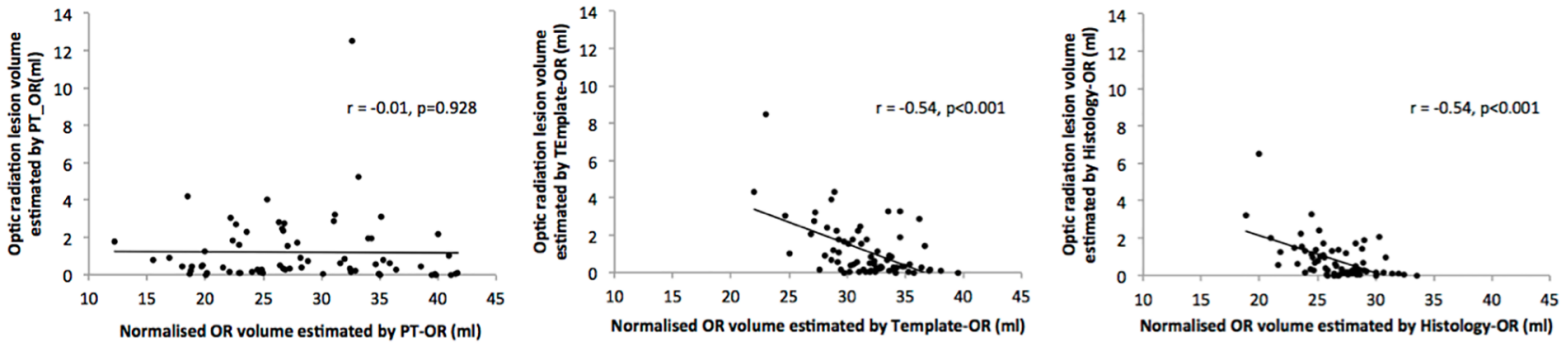
Correlations between OR volumes and OR lesion volumes.

As shown in Fig 8, there were significant correlations between normalised OR volume and OR lesion volume for Template-OR (r = -0.54, p<0.001) and Histology-OR (r = -0.54, p<0.001), but not PT-OR (r = -0.011, p = 0.928). The correlation coefficient of whole brain lesion and normalised brain volume was -0.67 (p<0.001).

### Optic radiation diffusivity estimation in subjects with MS

**Fig 9:**
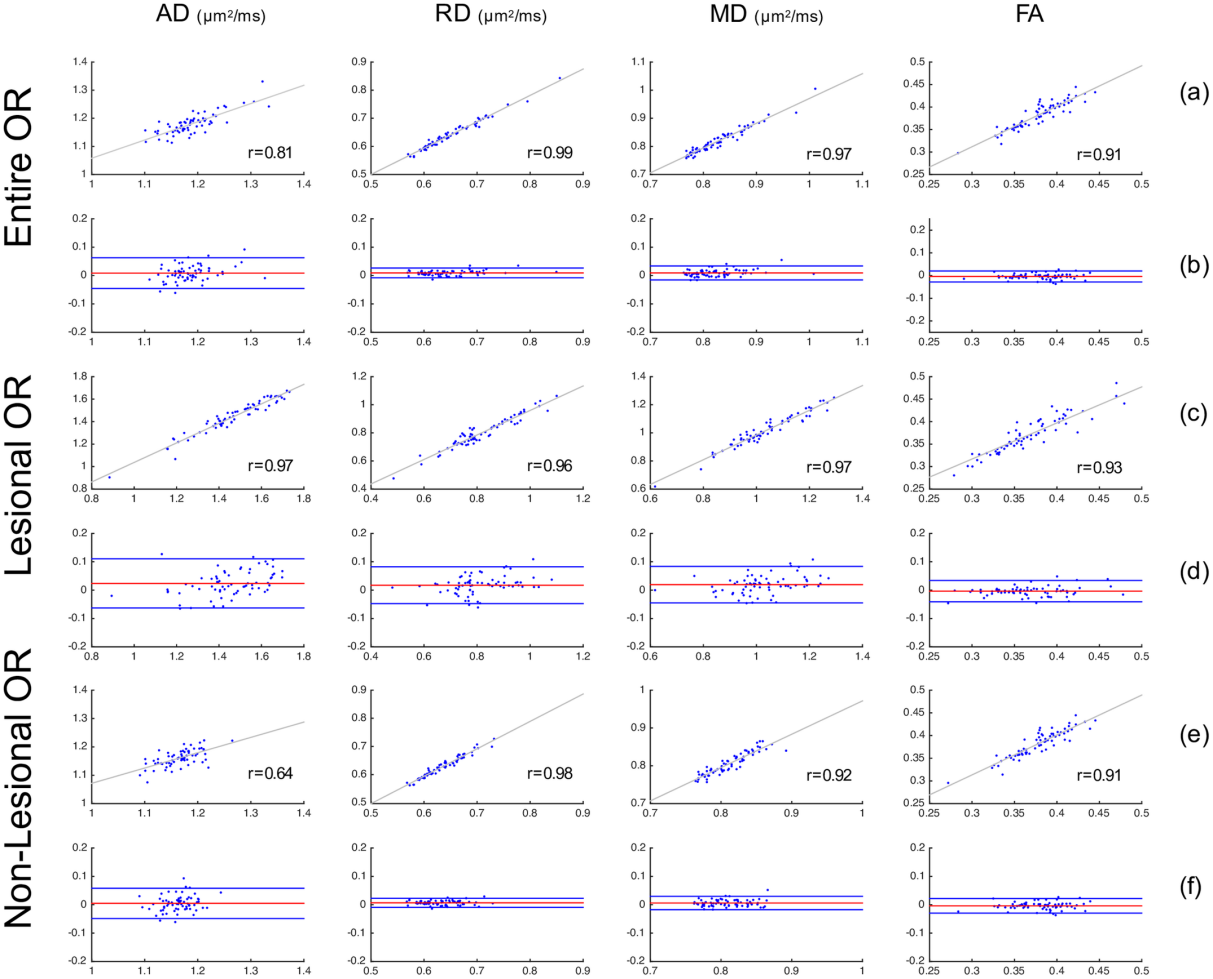
Comparison of OR diffusivity metrics between PT-OR and Template-OR approaches. Row (a), (c), (e) are scatter plots with linear fitting; x-axis is PT-OR measurement and y-axis is Template-OR measurement. r is the Pearson’s correlation coefficient. Row (b), (d), (f) are Bland-Altman plots for comparing two methods. x-axis is the mean of PT-OR and Template-OR measurements. y-axis is the difference between PT-OR and Template-OR measurements.

**Fig 10:**
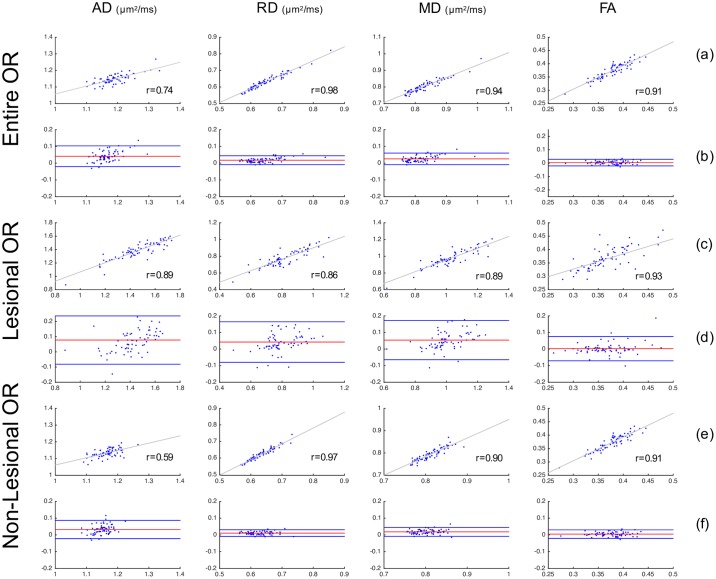
Comparison of OR diffusivity metrics between PT-OR and Histology-OR approaches. Row (a), (c), (e) are scatter plots with linear fitting; x-axis is PT-OR measurement and y-axis is Histology-OR measurement. r is the Pearson’s correlation coefficient. Row (b), (d), (f) are Bland-Altman plots for comparing two methods. x-axis is the mean of PT-OR and Histology-OR measurements. y-axis is the difference between PT-OR and Histology-OR measurements.

**Fig 11:**
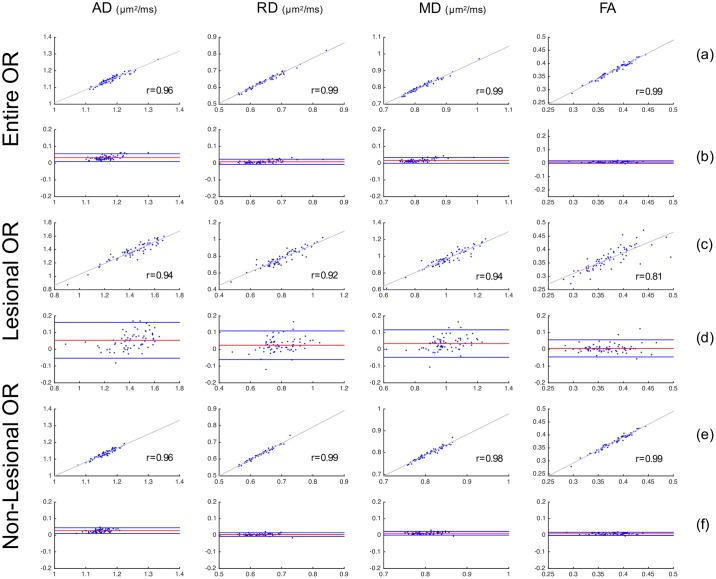
Comparison of OR diffusivity metrics between Template-OR and Histology-OR approaches. Row (a), (c), (e) are scatter plots with linear fitting; x-axis is Template-OR measurement and y-axis is Histology-OR measurement. r is the Pearson’s correlation coefficient. Row (b), (d), (f) are Bland-Altman plots for comparing two methods. x-axis is the mean of Template-OR and Histology-OR measurements. y-axis is the difference between Template-OR and Histology-OR measurement.

Table 2 summarises the average of diffusivity indices (AD, RD, MD and FA) in the entire OR, lesional OR and non-lesional OR. There was generally a good agreement between OR diffusivity measures derived using all three reconstruction techniques (Table 3). There was no significant difference between three OR techniques for all diffusivity measurements, with the exception of AD and MD for PT-OR vs Histology-OR in all the three OR measurement categories, between Template-OR and Histology-OR for the entire OR AD and between Template-OR and Histology-OR for non-lesional AD/MD (Table 3). Diffusivity differences between different OR segmentation techniques were consistently small across low and high values. Detailed evaluation of statistical precision and accuracy between different methods, including linear regression and Bland-Altman plots, is provided in Figs 9, 10 and 11.

**Table 2:** OR diffusivity measurement.

	PT-OR			Template-OR			Histology-OR		
	OR	L	NL	OR	L	NL	OR	L	NL
Mean AD (SD) ×10^−3^mm^2^/s	1.19 (0.047)	1.46 (0.171)	1.17 (0.034)	1.18 (0.038)	1.44 (0.154)	1.16 (0.029)	1.15 (0.030)	1.38 (0.133)	1.13 (0.025)
Mean RD (SD) ×10^−3^mm^2^/s	0.65 (0.054)	0.82 (0.120)	0.63 (0.038)	0.64 (0.052)	0.80 (0.109)	0.63 (0.038)	0.63 (0.047)	0.78 (0.096)	0.62 (0.037)
Mean MD (SD) ×10^−3^mm^2^/s	0.83 (0.049)	1.03 (0.129)	0.81 (0.030)	0.82 (0.045)	1.01 (0.117)	0.81 (0.029)	0.80 (0.039)	0.98 (0.102)	0.79 (0.028)
Mean FA (SD)	0.38 (0.030)	0.37 (0.050)	0.38 (0.031)	0.38 (0.030)	0.37 (0.044)	0.38 (0.030)	0.37 (0.030)	0.37 (0.041)	0.37 (0.030)

L: lesional OR; NL: non-lesional OR; AD: Axial Diffusivity; RD: Radial Diffusivity; MD: Mean Diffusivity; FA: Fractional Anisotropy

**Table 3:** Agreement between three methods in diffusivity measurement.

	r (PT-OR vs Template-OR)			r (PT-OR vs Histology-OR)			r (Template-OR vs Histology-OR)		
	OR	L	NL	OR	L	NL	OR	L	NL
AD	0.81	0.97	0.64	0.74	0.89	0.59	0.96	0.94	0.96
RD	0.99	0.96	0.98	0.98	0.86	0.97	0.99	0.92	0.99
MD	0.97	0.97	0.92	0.94	0.89	0.90	0.99	0.94	0.98
FA	0.91	0.93	0.91	0.91	0.93	0.91	0.99	0.81	0.99

L: lesional OR; NL: non-lesional OR; AD: Axial Diffusivity; RD: Radial Diffusivity; MD: Mean Diffusivity; FA: Fractional Anisotropy

Table 3 shows the strong correlation of diffusivity measurement among three methods (p<0.001), except relatively lower agreement between PT-OR vs Template-OR/Histology-OR for non-lesional OR AD.

## Discussion

In this study, we investigated the compatibility of tractography-based and template-based techniques with optimisation to delineate OR in MS patients and their potential impact on measurement of MS pathology in the posterior visual pathways. We enhanced previously described [30,31] analysis pipelines to yield outputs from both individual tractography and template-based analysis of the OR, while minimising the impact of focal MS lesions. To achieve this, 1) PT was used for superior performance in the presence of WM lesions and more realistic reconstruction of Meyer’s loop compared with DT; 2) non-linear co-registration of reference and subject scans was applied to maintain topographic integrity of WM structures and facilitate valid inter-subject comparison; 3) susceptibility-induced EPI distortion was corrected for accurate mapping of lesions and tractography seeding ROIs derived from FLAIR/T1weighted images to dMRI maps; 4) “lesion filling” was performed to reduce the impact of variation in lesion patterns on co-registration between standard template and individual subjects; and, 5) unlike previous methods [31], free of FA threshold was applied to avoid inadvertent exclusion of severely damaged tissues and inflamed oedematous lesions.

The main findings of the study may be summarised as follows:

The template-based approach yielded the largest OR volume with the smallest inter-subject variability. This approach also showed the highest correlation with NBV and total brain lesion volume, and displayed a relatively larger OR lesion load.

Conversely, individual probability-based tractography resulted in a significantly smaller mean OR volume that did not correlate with Template-OR or His-OR derived volumes. PT-OR volume was weakly associated with NBV and did not correlate with total brain lesion load.

These discrepancies are in part attributable to the particular methodology of OR delineation. For example, correlation between OR and brain volume is inherent to the template-based technique, since it is based on non-linear transformation of the OR template, which is in turn related to the shape and size of occipital lobe. Hence, the size of the projected OR is expected to be proportional to the degree of tissue loss both within the OR and surrounding WM structures. It is difficult to exclusively measure OR volume given that co-registration is based on T1/T2 structural imaging, where limited contrast between the OR and surrounding WM impedes the generation of accurate OR- specified deformations during non-linear co-registration. On the other hand, non-OR tissue loss has limited impact on the reconstruction of the OR by individual tractography based on WM tensors that provide contrast between OR and surrounding WM. Segmentation using PT-OR may, therefore, more accurately reconstruct individual ORs despite the manual fibre cleaning (an essential element of probabilistic tractography) and the effect of focal lesion-associated decreases in anisotropy inevitably causes higher variability of PT-derived OR volume.

The larger OR lesion volume derived by the template-based technique, which yields the largest OR volume, is also not surprising, since MS lesions have a periventricular predominance and commonly involve the OR.

Previous studies have compared tractography and template-based techniques to assess MS-related brain pathology. Lagana et al., [32] studied the corpus callosum in MS and concluded that individual tract-based (deterministic) analysis is more sensitive than atlas-based methods for measuring MS disability related structural changes, particularly in small cohorts. Reich et al., [31] applied a template-based method [30] to both healthy controls and patients with MS and yielded results comparable with those obtained using a conventional DT approach on corpus callosum, optic tract, corticospinal tract and OR. However, brain volume loss and dynamic ventricular enlargement in MS critically impacts the mapping of template-subject ROIs based on transformation matrices estimated by linear co-registration, the technique used in the Reich study [31]. Moreover, the FA thresholds chosen in this study (FA > 0.13 to streamline tractography inclusion criteria; FA < 0.25 to remove CSF partial volume contamination) could potentially ‘remove’ not only chronic destructive MS lesions, but also acute, severely inflamed edematous lesions.

A number of advanced diffusion tissue models have recently been applied to improve tractography outcomes in OR segmentation, with particular attention to the quality of Meyer’s loop reconstruction. Meyer’s loop comprises the inferior OR fascicles that abruptly change orientation at the temporal pole, thereby posing significant challenges for tractography algorithms. Thus, Kammen et. al [36] reported a fully-automated pipeline for OR reconstruction using fibre orientation distribution (FOD) [37,38] with high order spherical harmonics (SPHARM) on multi-shell high angular resolution diffusion imaging (HARDI) data from the Human Connectome Project (HCP) [39,40]. This pipeline generated Meyer’s loop reconstruction and retinotopic organisation of the OR consistent with a post-mortem dissection study [41]. Chamberland et al. developed a technique that employs additional ROIs based on anatomicallyexpected OR fibre orientation [42], and demonstrated superior delineation of Meyer’s loop compared with conventional tractography.

Therefore, new tractography algorithms potentially improve the performance of individual tract-based approaches. However, while sophisticated tractography delineates the OR with increased precision and accuracy, advanced pipelines may require demanding MRI acquisition techniques and complex tissue modelling, limiting their applicability to multicentre clinical trials in which MRI centre selection is primarily determined by the capacity of study sites to recruit subjects. For example, the multi-shell acquisitions used in the HCP would be difficult to deploy in a clinical environment.

One major advantage of template-based WM segmentation is that it reduces the minimum acquisition requirements for performing tract-specific analysis and may even be applied to datasets with no dMRI sequences (or with sub-optimal diffusion acquisitions including retrospective analyses). Template-based WM tract segmentation is likely to require significantly less image analysis expertise and computational power than individual tractography-based analysis. Typically, it includes following steps: data format conversion, brain extraction, nonlinear registration and template mapping. Additional steps for MS images include lesion analysis and lesion filling. The pipeline is highly automatable and particularly applicable to large datasets. In the present study, we have specifically addressed concerns regarding the reliability of using the Jülich histological atlas for template-based mapping and tract specific analysis.

Conversely, individual tractography-based methods typically require more complex pipelines and involve extensive manual QA when compared with template-based OR reconstruction. In general, the image processing pipeline includes the following steps: data format conversion, dMRI pre-processing (Eddy current, movement and EPI susceptibility distortion correction), tensor reconstruction, rigid co-registration with T1/T2 weighted images, placement of seeding ROIs and tractography followed by manual fibre cleaning. Although fully automated methods have been developed for seeding ROIs and tractography [36], close quality assurance with expert knowledge of anatomy and neuroimaging is imperative. Significant variations in pathology, such as NBV and focal white matter lesions, further challenge automated tractography algorithms and limit their applicability to diseases such as MS. Despite this, individual tractography-based methods remain valuable. Thus, while template-based methods present WM structures as a cluster of voxels devoid of the fibre “pathway” information (ROI-type analysis), fibre-based tractography provides connectivity information, and facilitates the construction of diffusivity profiles [43] along fibre bundles. Additionally, in conditions characterised by localised brain pathology, fibre-based tractography permits separation of the OR into ‘lesional’ and ‘non-lesional’ fibres, as defined by the presence or absence of lesions at any point along specific OR fibres, to quantitate tissue damage beyond but also relevant to focal T2 lesions in applications such as the analysis of Wallerian and retrograde neurodegeneration [44,45]. Both mechanisms of degeneration potentially contribute to progressive irreversible tissue loss in MS [44,46,47]. ‘Lesional’ fibres can be further separated into those traversing the lesion, proximal to the lesion (between cell body and lesion, to study retrograde neurodegeneration) and distal to the lesion (between lesion and axon terminal, to study Wallerian neurodegeneration) [44]. “non-lesional” fibres, on the other hand, identify normal appearing white matter that is unrelated to distant lesions, while maintaining topological conformity with adjacent lesional fibresreducing inter- and intra-subject variability[44,48].

A potential approach that combines the merits inherent to both methods could include non-linear OR template mapping using deformation matrices derived from non-linear co-registration between WM tensors (or the OR FA map), rather than T1/T2 structural images. This hypothetical pipeline may avoid the complexity of tractography and its inherently high variance, while maintaining the integrity of WM tract structure that is lost when segmentation is attempted using T1/T2 structural images. However, tools to interpolate the tensors within focal WM lesions and remove their impact on non-linear co-registration (analogous to lesion-filling tools for T1 structural images) are yet to be developed. Additionally, the application of free water elimination tools [49], which remove the isotropic component from lesions, may further reduce the impact of lesions on tensor based co-registrations.

## Conclusion

The application of ConTrack probabilistic tractography and template-based OR reconstruction techniques in subjects with MS yields comparable OR lesion volumes and diffusivity measurements, despite differences in the OR volume estimation. The choice of OR reconstruction technique should be determined primarily by the research question and the nature of the available dataset. Template-based approaches are particularly suited to the semi-automated analysis of large image datasets and have utility even in the absence of dMRI acquisitions. Individual tractography methods, while more complex than template-based OR reconstruction, permit measurement of the diffusivity profile along fibre bundles that are affected by specific MS lesions or other focal pathology.

## Supporting information

S1 File Optic radiation template. (ZIP)
